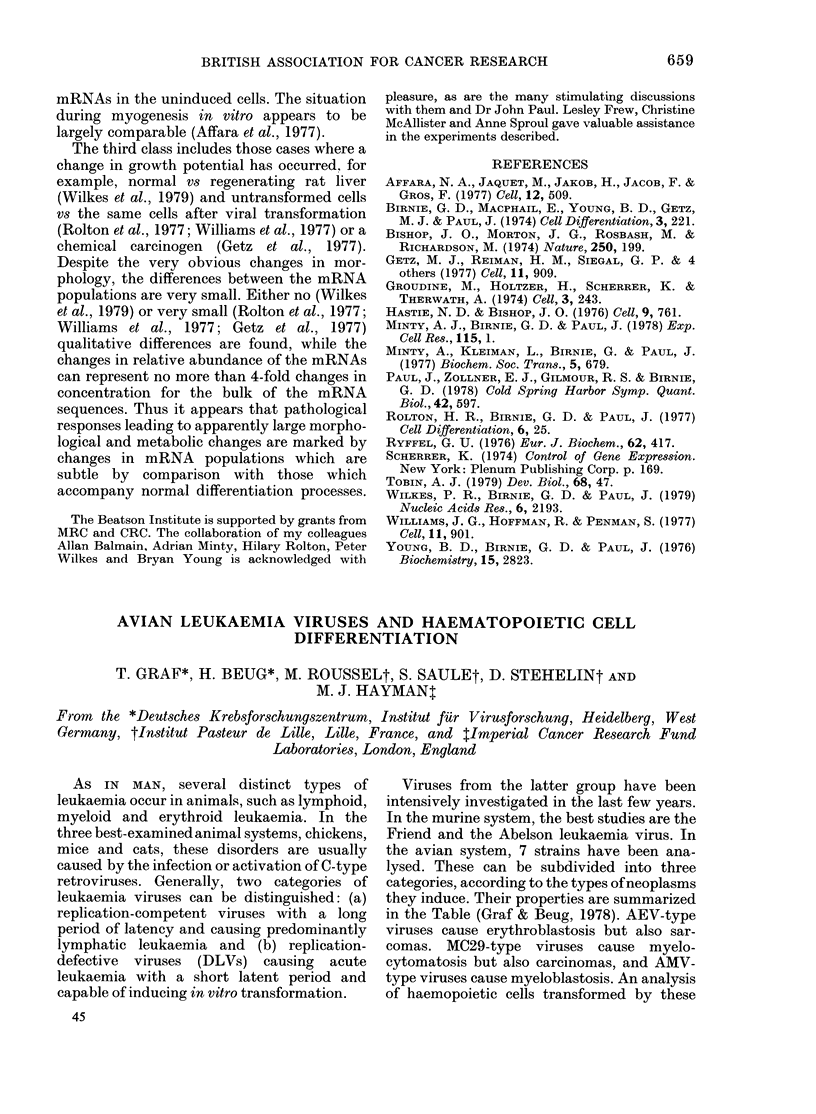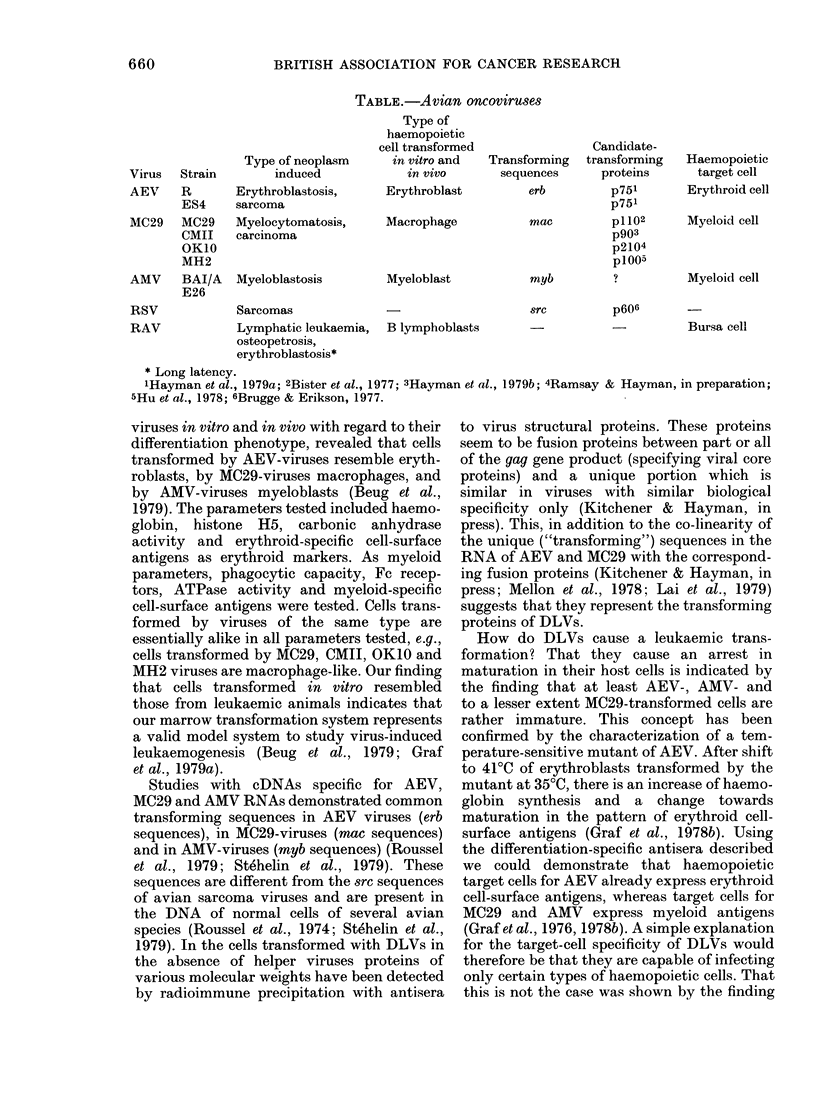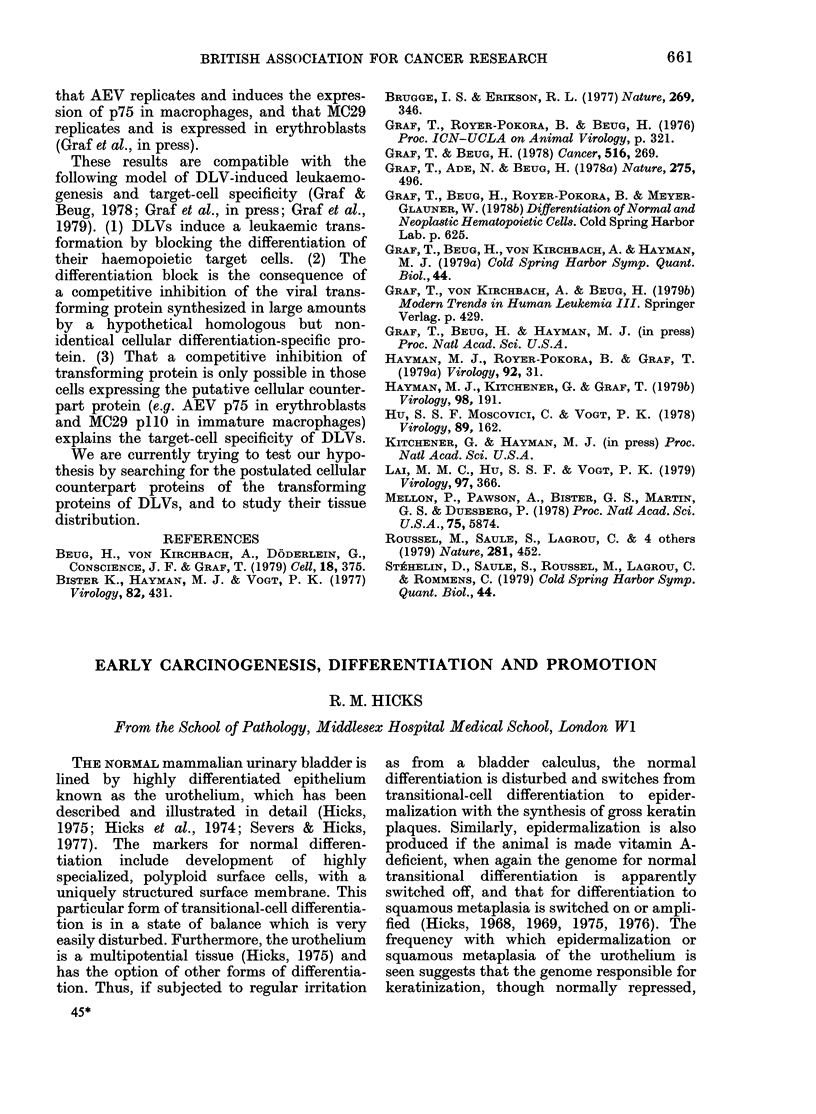# Avian leukaemia viruses and haematopoietic cell differentiation.

**DOI:** 10.1038/bjc.1980.114

**Published:** 1980-04

**Authors:** T. Graf, H. Beug, M. Roussel, S. Saule, D. Stehelin, M. J. Hayman


### AVIAN LEUKAEMIA VIRUSES AND HAEMATOPOIETIC CELL DIFFERENTIATION

T. GRAF*, H. BEUG*, M. ROUSSEL†, S. SAULE†, D. STEHELIN† and M. J. HAYMAN‡

*From the *Deutsches Krebsforschungszentrum, Institut für Virusforschung, Heidelberg, West Germany, †Institut Pasteur de Lille, Lille, France, and ‡Imperial Cancer Research Fund Laboratories, London, England*

As in man, several distinct types of leukaemia occur in animals, such as lymphoid, myeloid and erythroid leukaemia. In the three best-examined animal systems, chickens, mice and cats, these disorders are usually caused by the infection or activation of C-type retroviruses. Generally, two categories of leukaemia viruses can be distinguished: (a) replication-competent viruses with a long period of latency and causing predominantly lymphatic leukaemia and (b) replication-defective viruses (DLVs) causing acute leukaemia with a short latent period and capable of inducing *in vitro* transformation.

Viruses from the latter group have been intensively investigated in the last few years. In the murine system, the best studies are the Friend and the Abelson leukaemia virus. In the avian system, 7 strains have been analysed. These can be subdivided into three categories, according to the types of neoplasms they induce. Their properties are summarized in the Table (Graf & Beug, 1978). AEV-type viruses cause erythroblastosis but also sarcomas. MC29-type viruses cause myelocytomatosis but also carcinomas, and AMV-type viruses cause myeloblastosis. An analysis of haemopoietic cells transformed by these

TABLE.—*Avian oncoviruses*

| Virus | Strain | Type of neoplasm induced | Type of haemopoietic cell transformed *in vitro* and *in vivo* | Transforming sequences | Candidate-transforming proteins | Haemopoietic target cell |
|---|---|---|---|---|---|---|
| AEV | R<br>ES4 | Erythroblastosis, sarcoma | Erythroblast | *erb* | p75[1]<br>p75[1] | Erythroid cell |
| MC29 | MC29<br>CMII<br>OK10<br>MH2 | Myelocytomatosis, carcinoma | Macrophage | *mac* | p110[2]<br>p90[3]<br>p210[4]<br>p100[5] | Myeloid cell |
| AMV | BAI/A<br>E26 | Myeloblastosis | Myeloblast | *myb* | ? | Myeloid cell |
| RSV | | Sarcomas | — | *src* | p60[6] | — |
| RAV | | Lymphatic leukaemia, osteopetrosis, erythroblastosis* | B lymphoblasts | — | — | Bursa cell |

* Long latency.
[1]Hayman *et al.*, 1979*a*; [2]Bister *et al.*, 1977; [3]Hayman *et al.*, 1979*b*; [4]Ramsay & Hayman, in preparation; [5]Hu *et al.*, 1978; [6]Brugge & Erikson, 1977.

viruses *in vitro* and *in vivo* with regard to their differentiation phenotype, revealed that cells transformed by AEV-viruses resemble erythroblasts, by MC29-viruses macrophages, and by AMV-viruses myeloblasts (Beug *et al.*, 1979). The parameters tested included haemoglobin, histone H5, carbonic anhydrase activity and erythroid-specific cell-surface antigens as erythroid markers. As myeloid parameters, phagocytic capacity, Fc receptors, ATPase activity and myeloid-specific cell-surface antigens were tested. Cells transformed by viruses of the same type are essentially alike in all parameters tested, *e.g.*, cells transformed by MC29, CMII, OK10 and MH2 viruses are macrophage-like. Our finding that cells transformed *in vitro* resembled those from leukaemic animals indicates that our marrow transformation system represents a valid model system to study virus-induced leukaemogenesis (Beug *et al.*, 1979; Graf *et al.*, 1979*a*).

Studies with cDNAs specific for AEV, MC29 and AMV RNAs demonstrated common transforming sequences in AEV viruses (*erb* sequences), in MC29-viruses (*mac* sequences) and in AMV-viruses (*myb* sequences) (Roussel *et al.*, 1979; Stéhelin *et al.*, 1979). These sequences are different from the *src* sequences of avian sarcoma viruses and are present in the DNA of normal cells of several avian species (Roussel *et al.*, 1974; Stéhelin *et al.*, 1979). In the cells transformed with DLVs in the absence of helper viruses proteins of various molecular weights have been detected by radioimmune precipitation with antisera to virus structural proteins. These proteins seem to be fusion proteins between part or all of the *gag* gene product (specifying viral core proteins) and a unique portion which is similar in viruses with similar biological specificity only (Kitchener & Hayman, in press). This, in addition to the co-linearity of the unique ("transforming") sequences in the RNA of AEV and MC29 with the corresponding fusion proteins (Kitchener & Hayman, in press; Mellon *et al.*, 1978; Lai *et al.*, 1979) suggests that they represent the transforming proteins of DLVs.

How do DLVs cause a leukaemic transformation? That they cause an arrest in maturation in their host cells is indicated by the finding that at least AEV-, AMV- and to a lesser extent MC29-transformed cells are rather immature. This concept has been confirmed by the characterization of a temperature-sensitive mutant of AEV. After shift to 41°C of erythroblasts transformed by the mutant at 35°C, there is an increase of haemoglobin synthesis and a change towards maturation in the pattern of erythroid cell-surface antigens (Graf *et al.*, 1978*b*). Using the differentiation-specific antisera described we could demonstrate that haemopoietic target cells for AEV already express erythroid cell-surface antigens, whereas target cells for MC29 and AMV express myeloid antigens (Graf *et al.*, 1976, 1978*b*). A simple explanation for the target-cell specificity of DLVs would therefore be that they are capable of infecting only certain types of haemopoietic cells. That this is not the case was shown by the finding that AEV replicates and induces the expression of p75 in macrophages, and that MC29 replicates and is expressed in erythroblasts (Graf *et al.*, in press).

These results are compatible with the following model of DLV-induced leukaemogenesis and target-cell specificity (Graf & Beug, 1978; Graf *et al.*, in press; Graf *et al.*, 1979). (1) DLVs induce a leukaemic transformation by blocking the differentiation of their haemopoietic target cells. (2) The differentiation block is the consequence of a competitive inhibition of the viral transforming protein synthesized in large amounts by a hypothetical homologous but non-identical cellular differentiation-specific protein. (3) That a competitive inhibition of transforming protein is only possible in those cells expressing the putative cellular counterpart protein (*e.g.* AEV p75 in erythroblasts and MC29 p110 in immature macrophages) explains the target-cell specificity of DLVs.

We are currently trying to test our hypothesis by searching for the postulated cellular counterpart proteins of the transforming proteins of DLVs, and to study their tissue distribution.

REFERENCES

Beug, H., von Kirchbach, A., Döderlein, G., Conscience, J. F. & Graf, T. (1979) *Cell*, **18**, 375.

Bister K., Hayman, M. J. & Vogt, P. K. (1977) *Virology*, **82**, 431.

Brugge, I. S. & Erikson, R. L. (1977) *Nature*, **269**, 346.

Graf, T., Royer-Pokora, B. & Beug, H. (1976) *Proc. ICN–UCLA on Animal Virology*, p. 321.

Graf, T. & Beug, H. (1978) *Cancer*, **516**, 269.

Graf, T., Ade, N. & Beug, H. (1978*a*) *Nature*, **275**, 496.

Graf, T., Beug, H., Royer-Pokora, B. & Meyer-Glauner, W. (1978*b*) *Differentiation of Normal and Neoplastic Hematopoietic Cells.* Cold Spring Harbor Lab. p. 625.

Graf, T., Beug, H., von Kirchbach, A. & Hayman, M. J. (1979*a*) *Cold Spring Harbor Symp. Quant. Biol.*, **44**.

Graf, T., von Kirchbach, A. & Beug, H. (1979*b*) *Modern Trends in Human Leukemia III.* Springer Verlag. p. 429.

Graf, T., Beug, H. & Hayman, M. J. (in press) *Proc. Natl Acad. Sci. U.S.A.*

Hayman, M. J., Royer-Pokora, B. & Graf, T. (1979*a*) *Virology*, **92**, 31.

Hayman, M. J., Kitchener, G. & Graf, T. (1979*b*) *Virology*, **98**, 191.

Hu, S. S. F. Moscovici, C. & Vogt, P. K. (1978) *Virology*, **89**, 162.

Kitchener, G. & Hayman, M. J. (in press) *Proc. Natl Acad. Sci. U.S.A.*

Lai, M. M. C., Hu, S. S. F. & Vogt, P. K. (1979) *Virology*, **97**, 366.

Mellon, P., Pawson, A., Bister, G. S., Martin, G. S. & Duesberg, P. (1978) *Proc. Natl Acad. Sci. U.S.A.*, **75**, 5874.

Roussel, M., Saule, S., Lagrou, C. & 4 others (1979) *Nature*, **281**, 452.

Stéhelin, D., Saule, S., Roussel, M., Lagrou, C. & Rommens, C. (1979) *Cold Spring Harbor Symp. Quant. Biol.*, **44**.